# Risk score for early prognostication of aseptic bone flap necrosis

**DOI:** 10.1007/s00701-024-06342-1

**Published:** 2024-11-18

**Authors:** Lennart Barthel, Susann Hetze, Oliver Gembruch, Mehdi Chihi, Marvin Darkwah Oppong, Yahya Ahmadipour, Philipp Dammann, Ulrich Sure, Ramazan Jabbarli

**Affiliations:** Department of Neurosurgery and Spine Surgery, Center for Translational Neuro- and Behavioral Sciences, University Hospital Essen, Hufelandstraße 55, 45147 Essen, Germany

**Keywords:** Aseptic bone flap necrosis, Biomarker, Bone flap reimplantation, Bone flap resorption, Decompressive hemicraniectomy, Metabolic bone disease

## Abstract

**Purpose:**

Aseptic bone flap necrosis (ABFN) is a common complication of autologous cranioplasty that often requires reoperation. This study aimed to create a risk score for ABFN using relevant demographic, clinical, and laboratory markers.

**Methods:**

We included all patients who underwent autologous cranioplasty after decompressive surgery between 2007 and 2019. We collected laboratory data, initial clinical diagnoses, and demographic parameters before autologous bone flap reimplantation. The significant predictors of ABFN identified in the final multivariate analysis were used to develop a risk score.

**Results:**

Of the 412 patients who underwent craniectomy, 58 (14%, 32 females: 55.2%) developed ABFN. The following independent predictors of ABFN were included in the risk score (0–7 points): craniectomy due to trauma or hemorrhagic stroke (2 points), younger age (< 40 years, 2 points), cranioplasty timing (> 95 days post-craniectomy, 1 point), glutamate-pyruvate transferase < 18 U/L (1 point), and serum creatinine level < 0.815 mg/dL (1 point). The ABFN rates in patients with scores of 0–2, 3–4, and 5–7 points were 4.2%, 16.1%, and 34.6%, respectively. The risk score demonstrated moderate diagnostic accuracy for predicting ABFN, with an area under the curve of 0.739.

**Conclusion:**

The proposed risk score may help in early identification of individuals prone to ABFN. These data suggest that future studies should investigate the significance of metabolic syndromes related to ABFN occurrence. Understanding the potential impact of metabolic factors on ABFN can enhance risk assessment and targeted preventive measures for patients undergoing cranioplasty procedures.

**Supplementary Information:**

The online version contains supplementary material available at 10.1007/s00701-024-06342-1.

## Introduction

Decompressive craniectomy (DC) is a standard neurosurgical procedure for treating intractable intracranial hypertension caused by traumatic brain injury, intracerebral, or subarachnoid hemorrhage, ischemic stroke, cerebral venous thrombosis, or tumors [2, 9, 17, 22, 23]. Cryoconservation of the bone flap and autologous reimplantation after neurological recovery are common procedures typically performed within 3 to 6 months after DC. Aseptic bone flap necrosis / resorption (ABFN) is a common problem [6], requiring a repeated cranioplasty surgery with an elaborately constructed artificial bone flap for implantation. This complication increases medical costs for patient treatment [4] and poses individual risks due to repeated general anesthesia and potential surgical complications. Risk factors for ABFN are still under discussion, but factors such as bone flap fragmentation, hydrocephalus, and young age are thought to be associated with ABFN [6]. The storage duration and type of storage at low temperatures are also considered to influence ABFN [15, 27]. Therefore, early identification of patients at risk of developing ABFN postoperatively is crucial for adapting treatment strategies.

This study aimed to identify laboratory biomarkers and demographic parameters predicting ABFN in patients after autologous bone flap implantation and to develop a score based on these potential risk factors in a large single-center study.

## Methods and materials

### Data management and study population

In this study, we included all patients who underwent autologous bone flap implantation after DC in our clinical department between 2007 and 2019. We only included patients who we had followed for at least four years to mitigate the potential impact of late-stage complications on study results.

We examined demographic data (age and sex), specific medical parameters, such as laboratory values, and the differences between these values before and after autologous bone flap implantation. Regarding sex and gender equity in research (SAGER): The hospital system only provided evidence of binary information for individual patients. The data gave no indication that a non-binary category has been a matter.

The following clinical data were additionally collected: type of diagnosis at DC, side (left, right, bilateral), time intervals (craniectomy to autologous bone flap implantation and autologous bone flap implantation to ABFN), and occurrence of post-hemorrhagic or postoperative hydrocephalus. Additionally, all other complications, apart from ABFN, after autologous bone flap implantation (e.g., bleeding, abscess, infection), were recorded.

### Operation and follow-up

As mentioned, DC has diverse indications, from acute trauma, such as subdural hematoma, fractures, and epidural hematoma, to conditions such as malignant medial infarcts or venous thrombosis. DC is also used to relieve intracranial pressure when conservative treatment fails.

After standard preoperative preparations, a reverse question mark incision was made. The temporalis muscle and scalp flap were retracted to expose the skull. Preoperative intravenous antibiotics were administered, and subcutaneous drains were placed post-procedure and left in for 24–48 h. The hemicraniectomy size was at least 12 cm, and the bone flap was preserved at -80 °C for future use without subcutaneous storage in any case.

The decision to reimplant the bone flap was made case-by-case, considering factors such as persistent brain swelling and the patient’s medical and neurological condition, or other factors like scar conditions and the overall treatment plan. Our neurosurgical team evaluated these factors and obtained consent before proceeding.

The bone flap was warmed in a 37 °C sodium chloride solution before reinsertion. The dural layer and bony margins of the skull defects were carefully dissected. The bone flap was then reinserted and secured using titanium plates and screws.

In cases where the defect still prolapsed above the bone margins or at the calvaria level and elevated positioning of the head did not suffice, lumbar drainage was placed one day preoperatively. In some cases, cerebrospinal fluid had to be punctured intraoperatively from the lateral ventricles (assisted by ultrasound).

**Fig. 1:**
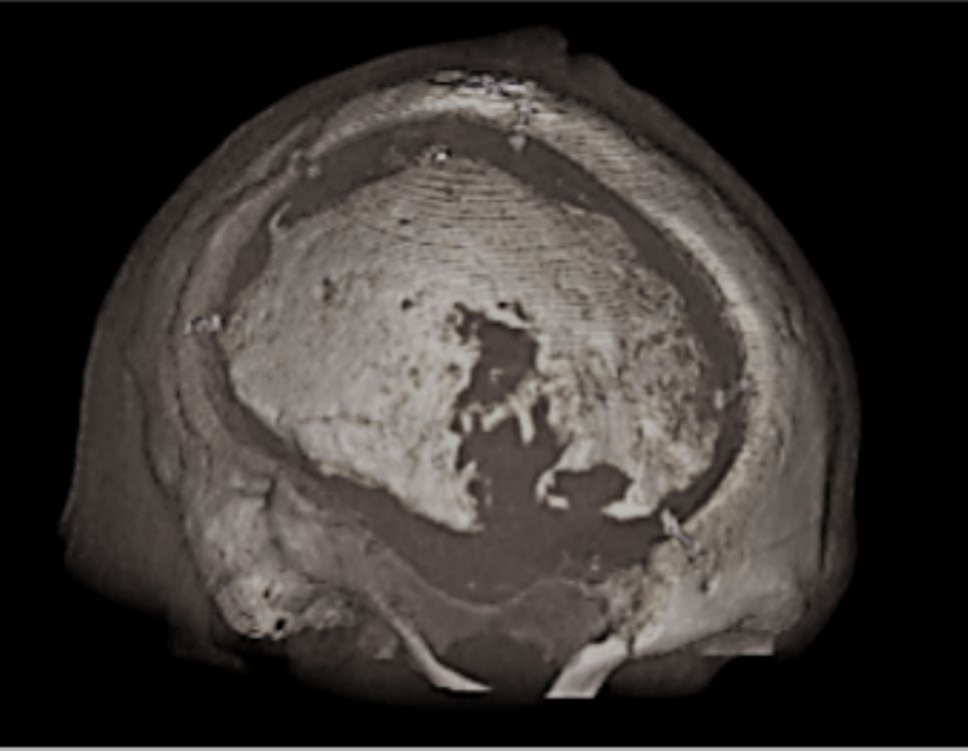
Computed tomography of aseptic bone flap necrosis (ABFN). Example case of aseptic bone flap necrosis; 3D reconstruction. Observed in a 51-year-old man who had undergone a right-sided hemicraniectomy due to an acute subdural hematoma and subsequent reimplantation of an autologous bone flap

All patients underwent postoperative computed tomography imaging within 24 h to detect bleeding, fluid effusions, or other complications. Subsequently, they were scheduled for follow-up appointments at our outpatient clinic, especially after completing rehabilitation and in cases of unusual events. In our study, we enrolled patients diagnosed with ABFN through clinical examinations and subsequent imaging or imaging alone (example for ABFN: Fig. 1).

### Statistical analysis and design of the risk score

The data were analyzed using IBM SPSS Version 21 (IBM Corporation, Armonk, New York, USA). P-values of less than 0.05 were considered statistically significant in the analyses. The goal of this study was to develop a risk score for ABFN prediction upon the parameters available at the time of initial DC (point in time 1 [PT1]) and autologous bone flap reinsertion (point in time 2 [PT2]). Accordingly, all parameters were tested in univariate and multivariate analysis to identify the most relevant ABFN predictors. First, laboratory values at PT1 and PT2 were analyzed as continuous variables using the unpaired t-test or Mann-Whitney test for normally and non-normally distributed parameters, as appropriate. Then, significant laboratory parameters and the remaining continuous parameters (age and timeframe between DC and cranioplasty) were dichotomized at the clinically relevant cutoffs for ABFN prediction according to the receiver operating characteristic (ROC) curve analysis. Diagnosis categories were dichotomized into two major groups: DC due to (traumatic/non-traumatic) hemorrhage and any other indication. Thereafter, the associations between the potential predictors (all in dichotomous manner) and ABFN were tested using the Fisher’s exact test. Significant results were then included in a final multivariate binary logistic regression analysis to determine independent associations with ABFN. Multiple imputation was used to replace the missing values.

Significant parameters identified through multivariate analysis were used to create a scoring system. The odds ratios (OR) of these parameters were divided by the smallest coefficient and rounded up to the nearest whole number to indicate their relative importance in risk score calculation. Each patient received a total point value based on the identified risk factors. The diagnostic accuracy of the novel risk score for ABFN was analyzed in the test cohort using the ROC curve. Additionally, the goodness of fit for the logistic regression model was assessed using the Hosmer-Lemeshow test.

### Ethical approval

The local Research Ethics Committee approved the study (proof number: 24-11849-BO). This study adheres to the Declaration of Helsinki regarding ethical principles for medical research involving humans, including research on identifiable human material and data, as well as the ICMJE recommendations for the conduct, reporting, editing, and publication of scholarly work in medical journals. As the analysis was anonymous, observational, and retrospective, informed consent was not required.

## Results

### Patient population and descriptive data

A total of 412 patients (median age: 51 years, range: 0–87 years; 187 females [45.4%] and 225 males [54.6%]) were included in this retrospective cohort study.

The most common incident that led to DC was an acute subdural hematoma (*n* = 169), followed by subarachnoid hemorrhage (*n* = 104). Table 1 lists all further diagnoses that led to DC, with information about the age distribution of the patients and how many of the patients developed ABFN with regard to the assigned diagnosis.

**Table 1:** Distribution of all patients included in this study (*n* = 412)

	Number of patients (*N*)	Percentage of all patients (%)	Females	Age ≤ 18 years	Age in years: mean / median [range]	Event of ABFN after autologous bone flap reimplantation
Acute disseminated encephalomyelitis	1	0.25	1	-	45	-
Acute subdural hematoma	127	31.3	34	5	54.8 / 57 [1–87]	18
Arteriovenous malformation	11	2.67	7	1	36.5 / 32 [14–62]	1
Brain edema	2	0.5	1	2	10 and 14	1
Brain tumor	24	5.83	14	3	48.25 / 52 [2–78]	1
Cerebral congestion (subsequent bleeding)	4	1	4	-	30, 48, 59, and 60	1
Empyema	1	0.24	1	1	14	-
Encephalitis	3	0.73	3	2	9, 12 and 23	-
Epidural hematoma	11	2.67	4	1	38.8 / 47 [3–65]	3
Gunshot injuries	2	0.5	-	-	25 and 32	-
Intracerebral bleeding	37	9.0	15	4	49.5 / 56 [0–75]	4
Malignant media infarct	58	14.1	29	1	46.5 / 47.5 [1–87]	4
Meningitis	3	0.73	-	-	29, 42, and 52	-
Subarachnoid hemorrhage (non-traumatic)	104	25.25	67	5	47.7 / 49.5 [0–74]	21
Subarachnoid hemorrhage (traumatic)	24	5.85	8	3	38.7 / 35 [13–69]	4
Total	412	100	188	28	51 [0–87]	58

The median age was 51 years (range: 0–87 years; 187 females [45.4%] and 225 males [54.6%]). All patients are listed according to the initial diagnosis leading to decompressive hemicraniectomy. The gender, age of the patients and how many of the respective diagnostic groups later developed an aseptic bone flap necrosis are indicated

*ABFN*Aseptic bone flap necrosis

In the postoperative course, chronic hydrocephalus requiring shunt placement was observed in 88 patients (21.4%) before (*n* = 17), simultaneously (*n* = 7), or after (*n* = 64) autologous cranioplasty. The bone flaps were stored for an average of 116.3 days (median: 99; range: 0–611; SD: 85.02) before reimplantation.

ABFN occurred in 58 patients (14.1%), with a slightly higher incidence in females (55.2%). The median time between bone flap reimplantation and diagnosis of ABFN was 2.1 years (mean: 771.2 days; median: 412.5 days; range: 23–3753 days).

### Risk factors for ABFN and the design of the score

In the initial screening of the eligibility of routine preoperative laboratory values before DC (PT1) and cranioplasty (PT2) for ABFN prediction (see Supplementary Table S1), gamma-glutamyl transferase (GPT) at PT1 (28.0 ± 25.1 vs. 40.2 ± 72.8 mg/dL, *p* = 0.02), glomerular filtration rate (GFR) at PT1 (106.3 ± 70.3 vs. 87.9 ± 36.2 ml/min, *p* = 0.034), and serum creatinine at PT2 (0.801 ± 0.221 vs. 0.882 ± 0.329 mg/dL, *p* = 0.05) were the only laboratory parameters significantly associated with the ABFN event. According to the results of ROC analysis, the following dichotomization for the selected continuous variables was performed: GPT at 18 U/L, GFR at 60 ml/min, creatinine at 0.815 mg/dL, age at 40 years, and the time between DC and cranioplasty at 95 days.

In the next step of uni*variate analysis *(Table 2)*, y*ounger age < 40 years old (odds ratio[OR] = 2.6, *p* = 0.001), DC due to intracranial hemorrhage (OR = 3.4, *p* = 0.004), GPT < 18 U/l at PT1 (OR = 2.4, *p* = 0.003), time between DC and cranioplasty > 95 days (OR = 1.83, *p* = 0.045), and serum creatinine < 0.851 mg/dl at PT2 (OR = 1.88, *p* = 0.032) were significant risk factors for ABFN. These five parameters were thereafter included in the final multivariate analysis (Table 3) and all confirmed as independent predictors of ABFN.

**Table 2:** Univariate analysis (Fisher’s test)

Parameter	OR (95% CI)	*p*-value
Parameters at the time of DC (PT1)		
Bleeding as initial diagnosis	3.4 (1.32–8.8)	**0.004**
Age < 40 years	2.6 (1.47–4.63)	**0.001**
Sex (female)	1.6 (0.9–2.8)	0.07
DC type (bifrontal craniectomy)	1.4 (0.60–3.11)	0.29
GPT < 18 U/L	2.4 (1.34–4.3)	**0.003**
GFR < 60 (ml/min)	0.52 (0.15–1.7)	0.21
Parameters at the time of cranioplasty (PT2)		
Time between DC and cranioplasty > 95 days	1.83 (1.01–3.32)	**0.045**
Creatinine at TP2 < 0.815 (mg/dl)	1.88 (1.06–3.34)	**0.032**
Ventriculoperitoneal shunt before cranioplasty	1.6 (0.89–3.1)	0.08

The significant p-values are highlighted in bold

*CI *Confidence intervals, *DC *Decompressive hrmicraniectomy, *dl *deciliter, *g *gram, *GFR *Glomerular filtration rate, *GOT *Glutamate–oxaloacetate-transaminase, *l *liter, *min *minutes, *ml *milliliters, *mmol *thousandth mole, *nl *nanoliters, *OR* odds ratio, *PT1* point of time 1 (before decompressive hemicraniectomy), *PT2* point of time 2 (before autologous bone flap reimplantation), *u* unit

**Table 3:** Risk score

Parameters	aOR (95% CI)	*P*-value	Score weight
Age at PT1 < 40 (years)	3.08 (1.67–5.67)	0.001	2
Bleeding as an initial diagnosis	4.60 (1.7–12.42)	0.003	2
Time between DC and cranioplasty > 95 (days)	2.13 (1.15–3.95)	0.016	1
GPT at PT1 < 18 (U/l)	2.19 (1.18–4.04)	0.013	1
Creatinine at PT2 < 0.815 mg/dl	1.98 (1.08–3.62)	0.027	1

Multivariate analysis of statistically significant predictors of aseptic bone flap necrosis (ABFN). Independent ABFN parameters identified in the univariate analyses were proven in the multivariate analysis. The table lists the parameters that were significant in the multivariate analysis and could thus be included in the score

*aOR* adjusted odds ratio, *CI* confidence interval. *DC* decompressive craniectomy, *dl* deciliter, *GPT* glutamate-Pyruvate-Transaminase, *l* liters, *mg* milligrams, *PT1* point of time 1 (before decompressive craniectomy), *PT2* point of time 2 (before autologous bone flap reimplantation), *p-value* probability of obtaining test results, *U *units

As described in the methods section, all independent ABFN predictors were used to construct a novel ABFN risk score. According to the aOR values from the multivariate analysis, score weights were calculated for each score component. So, age < 40 years (aOR = 3.08, *p* < 0.0001) and intracranial bleeding as initial diagnosis (aOR = 4.60, *p* = 0.003) were assigned **2 points**. The remaining score components were assigned **1 point** each: >95 days between DC and cranioplasty (aOR = 2.13, *p* = 0.016), GPT < 18 U/L at PT1 (aOR = 2.19, *p* = 0.013), and serum creatinine < 0.815 mg/dL at PT2 (aOR = 1.98, *p* = 0.027). The resulting novel risk score for ABFN prediction ranged between 0 and 7 points.

**Fig. 2:**
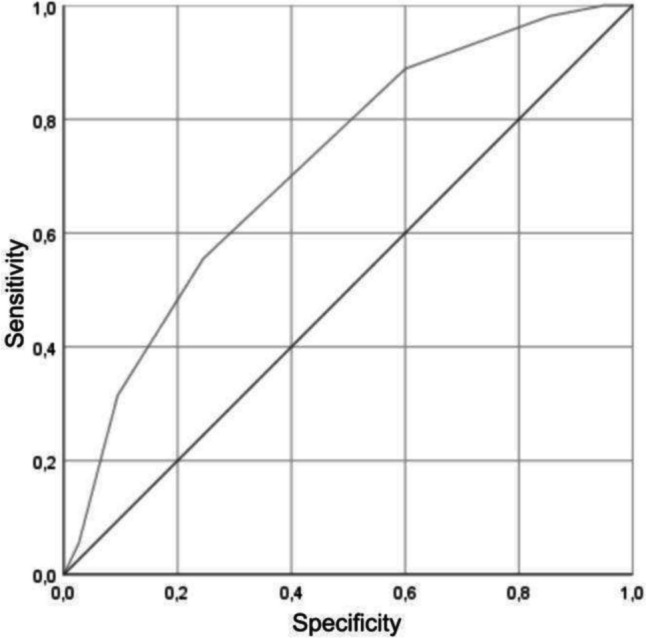
ROC curve (receiver operating characteristic) for the aseptic bone flap resorption score. Area under the curve: 0.739 (standard deviation: 0.36; asymptotic 95% confidence interval: 0.648–0.787)

The score values were then calculated for all patients. The mean score value in the cohort was 3.4 points (median: 3.0 points, range: 0–7 points). The ROC curve analysis (Fig. 2) showed a moderate diagnostic accuracy of the novel risk score for ABFN prediction, with the area under the curve of 0.739 (*p* < 0.0001).

**Fig. 3:**
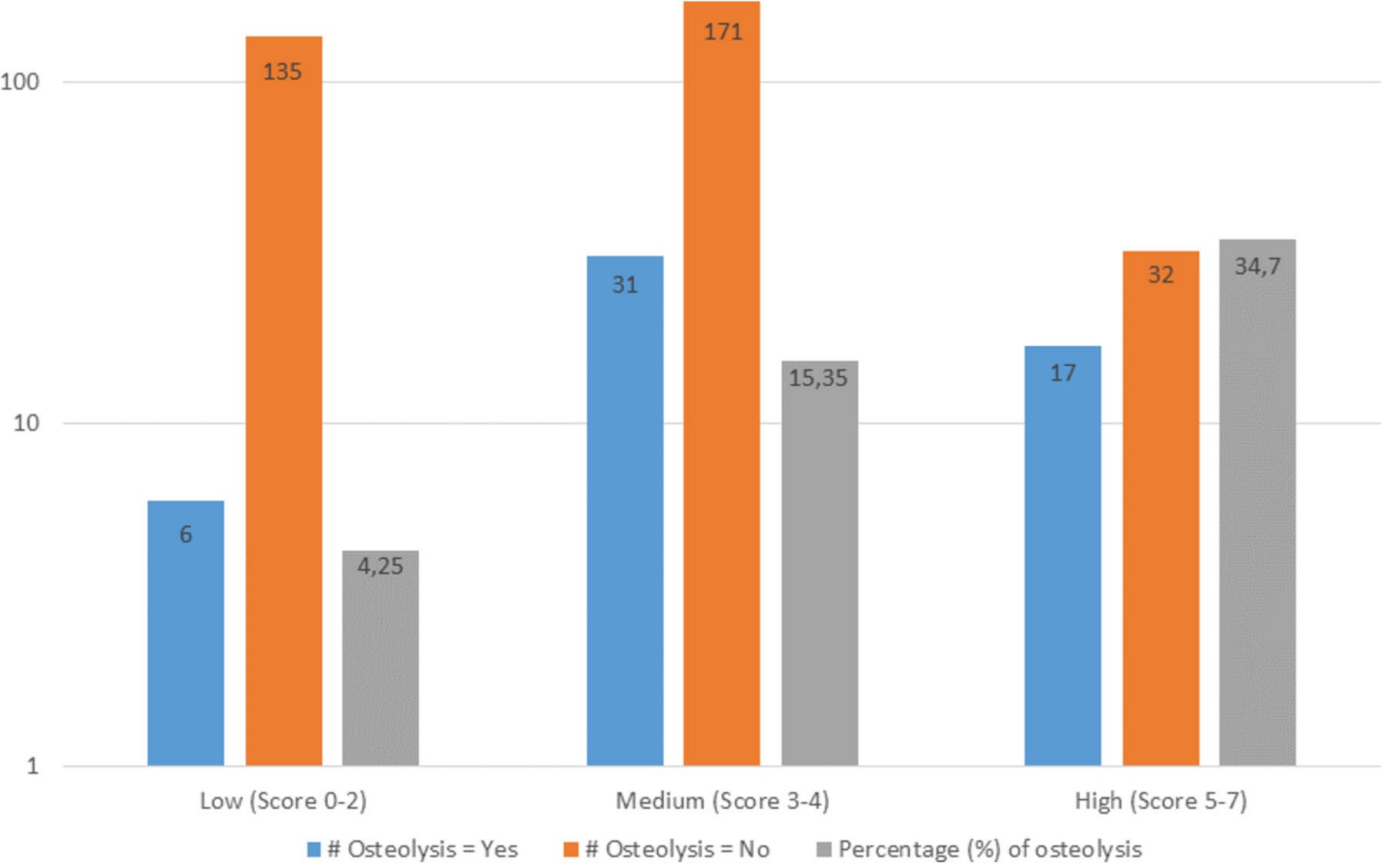
Distribution of the number of cases that were included in the risk score. All cases were included in the score after multivariate logistic regression analysis. The percentage of ABFN increased with higher scores (the y-axis is logarithmically shown)

To categorize the risk score into major risk levels, the risk points were divided into three groups: 0–2 points (low risk), 3–4 points (medium risk), and 5–7 points (high risk). The rates of ABFN gradually increased in these three major risk categories – 4.2%, 16.1%, and 34.6% in the low, medium and high risk groups respectively (Fig. 3).

## Discussion

The algorithms used in scoring systems manage individual patient clinical risks and aid in treatment decision-making, enhancing treatment predictions and options. Thus, they increase treatment efficiency. Here, we developed a score predicting ABFN by incorporating pre-cranioplasty demographic and laboratory data. Complications post-autologous bone flap implantation are frequent. ABFN definitions vary, resulting in significantly differing reported rates. For instance, rates between 23% and 30% were reported [6, 27], while others suggested lower rates ranging from 3–5.6% [18, 25]. However, the wide range in the occurrence of ABFN seems to indicate that there are no uniform assessment standards for the diagnosis of ABFN. Our study observed a 14% ABFN occurrence rate, aligning with the average range compared to other studies.

Furthermore, a score can help to estimate which patients may experience ABFN, considering factors such as implantation timing after DC (freezing storage time) of the bone flap and patient demographics, such as age. In addition to the factors discussed in the literature, our scores incorporated laboratory chemical markers, suggesting that specific metabolic conditions influence ABFN occurrence.

To our knowledge, no data exists on the association between laboratory values and ABFN development. We collected all available routine laboratory parameters before DC and autologous bone flap implantation to explore their potential association with ABFN. However, out of all examined values, two parameters, GPT and serum creatinine, showed independent associations with ABFN. Furthermore, nonlaboratory markers, such as the initial diagnosis and time between DC and cranioplasty, were associated with an ABFN risk.

Additionally, we observed a correlation between young age (< 40 years) and ABFN occurrence in our cohort, consistent with other studies [5, 6]. Some studies suggest increased markers for osteogenesis and osteoclastogenesis in younger patients (cancellous bone) [3], and it was found that there is a simultaneous occurrence of osteoblastic reintegration and necrosis with osteoclastic activity. This suggests that there is an imbalance in the intricate processes of bone integration (in necrotic skull bone flaps) [13] – the skull’s pronounced diploe provides ample space for potential bone remodeling activity. These findings suggest that bone resorption is a possible reason for this phenomenon, indicating a complex interplay between these processes in ABFN development. Understanding the underlying mechanisms of this bone metabolism imbalance is essential for gaining insights into ABFN pathogenesis and may lead to new targeted therapeutic approaches.

Elevated GPT levels can be an indicator for liver damage. In infants up to 12 months old, normal values can reach 59 U/L, gradually decreasing with age to less than 23 U/L for females and less than 29 U/L for males by age 18. For adult women, the reference range is typically < 34 U/L, and for men, < 45 U/L. In clinical routine, values of up to 31 U/L can be considered as normal for female children and adults and up to 41 U/L for male children and adults. [12, 20, 21] Our study found that a lower GPT levels, specifically below 18 U/L, predicted ABFN. Previous research linked low GPT levels to increased mortality risk in both elderly and mid-aged patients [8, 19, 24]. There is also a correlation between low GPT levels and increased circulating osteocalcin levels [11], the latter is observed in conditions which are characterized by loss of bone substance - such as osteoporosis [7]. Further studies could explore whether metabolic syndromes play a role in ABFN development.

Local inflammation is a common occurrence in cases of acute subdural hematoma, trauma, and stroke, as indicated by previous studies [28, 30]. Interestingly, this inflammatory state may persist beyond the acute phase and become a chronic condition in some instances. Chronic inflammation involves a prolonged immune response, which releases inflammatory mediators such as cytokines and chemokines. It is important to note that the immune system plays a significant role in bone remodeling. However, chronic inflammation can disrupt bone homeostasis, leading to an imbalance in the bone remodeling process and resulting in pathological bone resorption [29]. In our study, most patients experienced acute subdural hematoma before undergoing DC. The long-term effects of local inflammation, as discussed, may contribute, at least partially, to necrosis development. Furthermore, extended bone exposure to cold temperatures can exacerbate bone necrosis. Research suggests that cold temperatures adversely affect both cortical and trabecular bones, reducing bone minerals and hindering bone formation and the thawing process itself negatively affect the bone mineral density [26, 31]. Consequently, this could increase cell and nuclear areas, collagen disorganization, and osteocyte loss. Notably, these effects may intensify at even lower temperatures [1].

Low creatinine levels impact bone mineral density. Studies indicate that serum creatinine levels within the normal range are associated with higher mineral density in adolescents and the elderly with normal kidney function. In adolescents aged 12–19 years and in elderly high serum creatinine levels are associated with higher bone mineral density and accordingly lower serum creatinine levels are associated with reduced bone mineral density [10, 14, 16]. Fittingly, our evaluation revealed creatinine values below 0.815 mg/dL as an independent predictor of ABFN. Given also higher GFR values in individuals with ABFN in our univariate analysis, our results underscore the potential relevance of kidney function in the process of ABFN after autologous bone flap implantation.

Our discovery that laboratory chemical data influence bone flap necrosis, is promising for understanding the pathogenesis of ABFN. Thus, individual metabolic properties may play a role in ABFN occurrence, so that a person-specific decision can be made as to whether an autologous or artificial bone flap graft should be used. This could enable the provision of targeted therapies to counteract ABFN.

### Limitations

Achieving balanced data distribution in a large retrospective study presents challenges due to various factors. These include variations in laboratory parameters, differences in surgical approaches by multiple surgeons, and incomplete documentation. Determining the precise onset of bone flap necrosis is difficult, as it may commence before clinical or imaging evidence of ABFN emerges. Moreover, some patients included in the study may develop ABFN in the future, despite our minimum 4-year follow-up period. The average time between autologous bone flap implantation and ABFN occurrence was approximately 2.1 years. However, future necrosis development in the individual cases documented here remains possible. These complexities and uncertainties underscore the challenges and limitations of a retrospective setting.

## Conclusion

In this study, we devised a score to predict ABFN after autologous bone flap implantation, incorporating demographic data and laboratory values. The risk score could streamline surveillance post-cranioplasty for patients at considerable ABFN risk. These results imply connections between metabolic syndromes, chronic inflammation, and bone necrosis, highlighting their intricate interplay in ABFN development. They stress the importance of continuous risk assessment and hint at tailored therapeutic strategies based on individual metabolic properties.

## Supplementary Information

Below is the link to the electronic supplementary material.

ESM 1 (DOCX 99.4 KB)

## Data Availability

No datasets were generated or analysed during the current study.
