# Supplementary material for: Risk score for early prognostication of aseptic bone flap necrosis
Source: Acta Neurochir (Wien). 2024 Nov 18;166(1):461. doi: 10.1007/s00701-024-06342-1 (PMC11573868; doi:10.1007/s00701-024-06342-1)
Supplement: Supplementary file 1 — (DOCX 99.4 KB) [file 701_2024_6342_MOESM1_ESM.docx]

**Supplementary Table S1|** *Univariate analysis for laboratory values collected directly before craniectomy and directly before autologous bone flap reimplantation. Levels of significance are depicted in bold.*

*Abbreviations: an. = inorganic; AP = alkaline phosphatases; aPTT = partial thromboplastin time; CRP = C-reactive protein; dl = deciliter; g = gram; GFR = glomerular filtration rate; GGT = gamma-glutamyl transferase; GOT (ASAT) = aspartate aminotransferase; GPT (ALAT) = glutamate-pyruvate transaminase; INR = international normalized ratio; l = litre; LDH = lactate dehydrogenase; mmol = millimole; nl = nanoliters; p - value = probability of obtaining test results; PT1 = point of time 1 (before decompressive hemicraniectomy); PT2 = point of time 2 (before autologous bone flap reimplantation); S-creatinine = serum creatinine; SD = standard deviation; sec = seconds; TPZ = thromboplastin time; U = units*

| Laboratory values | Osteolysis = No | | Osteolysis = Yes | | |
| --- | --- | --- | --- | --- | --- |
|  | mean | SD | mean | SD | P - value |
| Preoperative laboratory values before decompressive hemicraniectomy (PT1) | | | | | |
| AP (U/l) | 71.6 | 29.1 | 70.85 | 28.3 | 0.901 |
| aPTT (sec) | 27.2 | 6.7 | 28.2 | 9.3 | 0.571 |
| Calcium (mmol/l) | 2.75 | 7.7 | 2.2 | 0.2 | 0.950 |
| CRP (mg/dl) | 2.5 | 4.1 | 2.2 | 3.95 | 0.464 |
| GFR (ml/min) | **87.9** | **36.15** | **106.25** | **70.3** | **0.034** |
| GGT (U/l) | 67.4 | 148.5 | 49.65 | 84.3 | 0.100 |
| GOT (ASAT) (U/l) | 49.05 | 94.05 | 37.25 | 43.2 | 0.259 |
| GPT (ALAT) (U/l) | **40.15** | **72.8** | **28** | **25.05** | **0.020** |
| Hemoglobin (g/dl) | 11.85 | 2.05 | 11.7 | 2.1 | 0.989 |
| INR | 1.1 | 0.4 | 1.05 | 0.25 | 0.249 |
| LDH (U/l) | 273.05 | 204.7 | 276.05 | 201.7 | 0.681 |
| Leukocytes / nl | 11.9 | 5 | 12.25 | 4.95 | 0.543 |
| Phosphate (an.) (mg/dl) | 3.5 | 0.95 | 3.4 | 0.9 | 0.842 |
| Potassium (mmol/l) | 4.2 | 0.45 | 4.2 | 0.5 | 0.509 |
| Protein (g/dl) | 6.2 | 1 | 6.05 | 1.2 | 0.815 |
| S-creatinine (mg/dl) | 1.2 | 4.3 | 0.85 | 0.25 | 0.120 |
| Sodium (mmol/l) | 140.45 | 4.8 | 140.65 | 5.8 | 0.393 |
| TPZ (Quick value) (%) | 0.95 | 0.2 | 0.95 | 0.2 | 0.683 |
| Uric acid (mg/dL) | 4.25 | 1.4 | 4.55 | 0.8 | 0.540 |
| Preoperative laboratory values before cranioplasty (PT2) | | | | | |
| AP (U/l) | 108.4 | 116.35 | 118.2 | 90.85 | 0.498 |
| aPTT (sec) | 108.4 | 116.35 | 118.2 | 90.85 | 0.498 |
| Calcium (mmol/l) |  |  |  |  |  |
| CRP (U/l) | 1.5 | 2.55 | 1.3 | 2.45 | 0.055 |
| GFR (ml/min) | 94.8 | 41 | 114.6 | 72.65 | 0.090 |
| GGT (U/l) | 75.05 | 182.65 | 63.2 | 124.05 | 0.093 |
| GOT (ASAT) (U/l) | 23.35 | 14.8 | 25.25 | 19.3 | 0.821 |
| GPT (ALAT) (U/l) | 35.55 | 33.5 | 39.15 | 48.05 | 0.535 |
| Hemoglobin (g/dl) | 12.45 | 1.9 | 12.55 | 1.9 | 0.541 |
| INR | 1 | 0.1 | 1 | 0.05 | 0.828 |
| LDH (U/l) | 196.85 | 58.8 | 202.95 | 60.3 | 0.679 |
| Leukocytes / nl | 7.7 | 2.85 | 7.3 | 2.8 | 0.263 |
| Phosphate (an.) (mg/dl) | 3.9 | 0.65 | 7.3 | 2.8 | 0.263 |
| Potassium (mmol/l) | 4.4 | 1.55 | 4.3 | 0.4 | 0.608 |
| Protein (g/dl) | 6.8 | 0.7 | 6.95 | 0.75 | 0.069 |
| S-creatinine (mg/dl) | 0.9 | 0.35 | 0.8 | 0.2 | 0.053 |
| Sodium (mmol/l) | 140.85 | 3.35 | 140.3 | 2.9 | 0.071 |
| TPZ (Quick value)(%) | 1 | 0.15 | 1 | 0.15 | 0.590 |
| Uric acid (mg/dL) | 5.05 | 1.65 | 4.5 | 1.6 | 0.115 |
